# Self-Reported Cultural Competence in Delivering Quality Care Among Primary Healthcare Physicians of Jeddah, Saudi Arabia: A Cross-Sectional Study

**DOI:** 10.7759/cureus.91348

**Published:** 2025-08-31

**Authors:** Ala A Aziz, Najlaa A Mandoura

**Affiliations:** 1 Preventive Medicine, Saudi Ministry of Health, Jeddah, SAU

**Keywords:** cultural competence, multicultural healthcare, physician training, primary healthcare, saudi arabia

## Abstract

Background

Cultural competence among healthcare providers is essential in delivering quality care within diverse populations. Saudi Arabia's healthcare system faces unique challenges due to its multicultural patient base. This study aimed to assess the levels of cultural competence among primary healthcare physicians at King Fahad General Hospital in Jeddah, Saudi Arabia.

Methods

A cross-sectional study was conducted among 85 primary healthcare physicians at King Fahad General Hospital (Jeddah, SAU). A validated cultural competence assessment tool, titled 'Residents' attitudes, perceived preparedness, and self-reported clinical practice behavior,' was used to evaluate three domains, namely understanding social and cultural context, recognizing cultural factors, and preparedness to address cultural issues. Data were analyzed using non-parametric tests (Mann-Whitney U and Kruskal-Wallis H) to examine associations between demographic variables and cultural competence scores, as the scores did not follow normal distribution.

Results

The highest competence was reported in recognizing cultural factors (median=86.6, interquartile range=27.2), followed by understanding social and cultural contexts (median=83.0, interquartile range=34.0), while preparedness to address cultural issues scored lowest (median=79.6, interquartile range=20.4). Among demographic factors, only specialty showed a significant association with cultural recognition (p=.02), with family medicine physicians scoring highest (mean rank=47.36), followed by general practitioners (mean rank=35.10).

Conclusion

Primary healthcare physicians at King Fahad General Hospital in Jeddah demonstrate strong awareness of cultural factors but are less prepared to address them in clinical practice. Targeted training, especially for specialties with lower recognition scores, is recommended to enhance practical cultural competence skills and improve healthcare delivery within Saudi Arabia’s multicultural environment.

## Introduction

Culture plays a central role in human psychological and psychosocial development, forming the foundation of societies [1]. Race, socioeconomic status, and health literacy significantly influence individuals' experiences within the healthcare system. These factors can impact patients' perceptions of symptoms and health, their decisions regarding when and how to seek medical care, their treatment preferences, and their adherence to prescribed medical plans [2].

Culturally competent healthcare is defined as “care that respects diversity in the patient population and cultural factors that can affect health and health care, such as language, communication styles, beliefs, attitudes, and behaviors” [3]. It is important to understand that cultural competence is not only linked to race and ethnicity but is also closely related to diversity competence, which is concerned with individuals with disabilities and other marginalized groups [3].

Cultural competence thus involves integrating cultural sensitivity into medical practice to improve patient outcomes and reduce disparities in healthcare. The core elements of cultural competence include cultural awareness, cultural knowledge, and cultural skill-development [4]. Cultural awareness involves understanding one's own cultural biases as well as understanding how these influence perceptions and interactions. It includes acknowledging potential unconscious biases and prejudices that can create barriers [5]. Cultural knowledge involves acquiring knowledge about different cultures and their health beliefs. Cultural skill development encompasses developing effective communication skills and engaging in cross-cultural interactions [4].

Culturally competent physicians possess “the ability to effectively deliver healthcare services that meet the social, cultural, and linguistic needs of patients” [6]. Culturally competent physicians can foster trust and understanding with patients from different cultural backgrounds by recognizing and adapting to their cultural norms and preferences. This can significantly impact treatment adherence and satisfaction [7]. A patient-centered approach requires an understanding of patients' cultural identities and how these identities influence health decisions. This ensures that treatment plans align with patients' cultural values. Cultural misunderstandings can hinder communication, lead to misdiagnosis, cause patients to not adhere to treatment, and result in poor health outcomes [8].

In order to bridge cultural gaps between healthcare providers and patients, physicians must acquire cultural competence by developing its three core elements. An earlier study found that positive attitudes toward cross-cultural care and experience serving diverse patient populations were significant predictors of perceived preparedness among internal medicine residents. However, knowledge of health disparities was relatively poor and did not vary significantly across different postgraduate training years [5].

Developing and maintaining cultural competence in healthcare is essential yet challenging. Language barriers, limited resources, and cultural misunderstandings can hinder effective communication and care delivery. However, cultural awareness training, immersion experiences, and inclusive practices can facilitate cultural competence. While cultural competence is important, some argue that a broader framework, such as cultural humility, is necessary. Cultural humility emphasizes ongoing learning and adapting to individual patient needs, thus addressing the complexities of cultural identity more effectively [9]. The Washington University Medical Plunge program and the Emergency Medicine Cultural Competency Curriculum are two initiatives that apply innovative approaches to address health disparities and improve patient care. The Washington University Medical Plunge incorporates a two-week diversity retreat that focuses on social determinants of health and cultural contexts. This retreat aims to enhance medical students' understanding of these factors. Similarly, the Emergency Medicine Cultural Competency Curriculum, which is required by the Accreditation Council for Graduate Medical Education, provides training for residents and faculty in cultural competence relevant to their patient populations [10,11].

In recent years, Saudi Arabia has experienced a significant influx of expatriates, creating a more diverse population within the kingdom. This shift in demographics necessitates a deeper understanding of cultural diversity and its implications for healthcare delivery [12]. Primary health care is the first level of health care service in Saudi Arabia and is provided by the Ministry of Health through a network of primary health care centers [12]. Primary healthcare in Saudi Arabia has gained significant focus as part of the Vision 2030 initiatives. Government agencies recognized the importance of assessing current activities in primary care units across various regions to understand the challenges faced by frontline staff and to evaluate how existing practices align with international best practice standards [13]. Primary healthcare physicians play a pivotal role in providing accessible, comprehensive healthcare services. Therefore, it is essential to assess their cultural competence to ensure high-quality care for all patients, regardless of their cultural background.

There is a notable lack of research focusing on cultural competence among primary healthcare physicians in Jeddah. A previous study in Jeddah, Saudi Arabia [14], revealed that physicians exhibit greater confidence in their clinical skills than in their intercultural communication abilities. This disparity underscores the scarcity of comprehensive data on physicians’ awareness and preparedness to engage effectively in culturally diverse clinical interactions. Therefore, it is imperative to conduct in-depth research to assess physicians’ cultural competence in Jeddah, identify existing gaps, and pinpoint areas requiring targeted improvement to enhance patient-centered care within this multicultural context.

Cultural competency is a vital aspect of patient-centered care, enabling providers to address diverse cultural needs effectively. However, it remains underexplored in Saudi Arabia’s healthcare system and requires further research to assess and enhance cultural competence among healthcare professionals in this multicultural context. Therefore, this study was conducted to assess cultural competence among primary healthcare physicians affiliated with King Fahad General Hospital (KFGH) in Jeddah. The objectives of this study included assessing physicians’ attitudes, perceived preparedness, and self-reported clinical practice behaviors in delivering care to culturally diverse patients in the primary healthcare setting.

## Materials and methods

Study design and population

An analytic cross-sectional study was conducted among physicians working in primary healthcare centers affiliated with KFGH in Jeddah, Saudi Arabia, between January and February 2025. The KFGH is a major healthcare institution that serves a diverse population of various socioeconomic backgrounds and nationalities.

Participants in the study included male and female physicians of any nationality working at primary healthcare centers affiliated with KFGH in Jeddah and who consented to participate. This study included a census of all eligible physicians working at the affiliated primary healthcare centers. Other healthcare providers such as dentists, nurses, pharmacists, and technicians were excluded, as the study focused specifically on physicians. Physicians on leave and those with less than one year of experience were excluded. At the time of data collection, no physician older than 55 years met the eligibility criteria in the selected centers.

Sample size calculation

The sample size was calculated using Cochran's formula [15] with a conservative prevalence estimate of 50% (since no prior data were available), a margin of error of 5%, and a confidence level of 95%. The formula used was n = (Z² * p * (1-p)) / e², where n is the sample size, Z is the Z-score (1.96 for a 95% confidence level), p is the estimated prevalence (0.5), and e is the margin of error (0.05). The p here represents the proportion of expected response. We have selected p as 0.5, as the variance of a proportion (p * (1-p)) is maximized when p = 0.5. Using 0.5 in the sample size formula ensures that the widest possible range of possible values is considered. By using 0.5, we are assuming the most uncertain scenario. The final sample size was 384. Although the calculated minimum sample size was 384, the total eligible population of physicians at the time of the study was limited. Accordingly, a census approach was adopted, and all eligible physicians meeting the inclusion criteria across the 12 affiliated primary healthcare centers (Al-Bawadi 1, Al-Bawadi 2, Al-Safaa 1, Al-Safaa 2, Al-Faisaliah, Al-Rehab, Al-Marwah, Al-Rabwah, Al-Naeem, Al-Slamah, Al-Nahdah, and Mushrefah) were invited to participate. Of these, 85 consented.

Data collection

Data were collected by a single researcher between January and February 2025 through face-to-face interviews with physicians using a standardized script. This research's aims and objectives were clearly explained to all participants, who then provided informed consent. Of the 86 eligible physicians approached, 85 consented to participate, yielding a response rate of 98.8%.

Data collection instrument

The study employed a pre-validated questionnaire that was designed by a previous study conducted on residents from a variety of specialties [5]. The questionnaire (see Appendix A) was originally validated in English, which is the working language of medical practice in Saudi Arabia; hence, no translation was required. It consists of four sections. Section one collected demographic information, including age, gender, nationality, qualification, specialty, and years of experience. Section two evaluated the participants' understanding of the social and cultural context. Section three measured beliefs regarding the importance of cultural diversity, awareness of unconscious biases, and self-assessed cultural sensitivity. Section four examined the participants’ readiness to provide care to patients from diverse backgrounds. Sections two to four were each scored on a scale from 0 to 100. Prior to full-scale data collection, a pilot test was conducted with 10 physicians to assess the clarity and feasibility of the questionnaire.

Ethical considerations

This study adheres to the tenets of the 1964 Declaration of Helsinki. The study received ethical clearance from the Institutional Review Board at the Directorate of Health in Jeddah (approval no. A02114). Informed consent was obtained from all participants. The questionnaire excluded sensitive or personal questions to ensure participant anonymity throughout the research process. Participant responses were anonymized at the point of data entry. All data were stored securely on password-protected computers accessible only to the researchers.

Statistical analysis

Data analysis was conducted using SPSS Statistics version 30 (IBM Corp., Armonk, NY, USA). Descriptive statistics were calculated for all variables. For demographic characteristics, frequencies and percentages were reported. For cultural competence scores, median values and interquartile ranges (IQR) were calculated due to the non-normal distribution of the data. Data distribution was assessed using the Shapiro-Wilk test, supported by visual inspection of histograms and Q-Q plots. As the cultural competence scores did not follow a normal distribution, nonparametric tests were employed for all analyses. The Mann-Whitney U test was used to compare cultural competence scores between two independent groups (gender and nationality), while the Kruskal-Wallis H test was utilized for comparing more than two independent groups (age categories, qualification levels, specialty types, and years of experience categories). For all statistical tests, a p-value less than 0.05 was considered statistically significant.

## Results

Demographic characteristics

Table 1 presents the demographic characteristics of the study participants. The majority of participants (52.9%) were between 25 and 35 years of age, while 37.6% were between 36 and 45 years, and 9.4% were between 46 and 55 years. There was a predominance of female physicians (64.7%) compared to males (35.3%). Most participants were Saudi nationals (95.3%), with only 4.7% being non-Saudi. Regarding qualifications, 45.9% of physicians were board-certified, 41.2% held bachelor's degrees, 7.1% had master's degrees, and 5.9% had subspecialty fellowships. Family medicine was the most common specialty (70.6%), followed by general practitioners (24.7%) and other specialties (4.7%). In terms of experience, most physicians (56.5%) had one to nine years of experience, 34.1% had 10 to 19 years, 8.2% had 20 to 29 years, and only 1.2% had 30 or more years of experience.

**Table 1:** Demographic characteristics (total n = 85)

Variables		N	Percentage
Age (in years)	25-35	45	52.9%
	36-45	32	37.6%
	46-55	8	9.4%
Gender	Male	30	35.3%
	Female	55	64.7%
Nationality	Saudi	81	95.3%
	Non-Saudi	4	4.7%
Qualification	Bachelors	35	41.2%
	Masters	6	7.1%
	Board certified	39	45.9%
	Subspecialty (fellowship)	5	5.9%
Specialty	General practitioner	21	24.7%
	Family medicine	60	70.6%
	Other specialty	4	4.7%
Years of experience	1-9	48	56.5%
	10-19	29	34.1%
	20-29	7	8.2%
	≥30	1	1.2%

Cultural competence

Table 2 shows the cultural competence scores across three dimensions. The recognition dimension had the highest median score (median=86.6, IQR=27.2). This was followed by the social and cultural context dimension (median=83.0, IQR=34.0). The preparedness dimension showed the lowest median score (median=79.6, IQR=20.4).

**Table 2:** Cultural competence among participants (total n=85) IQR: Interquartile range

Variables	Median	IQR
Social and cultural context	83.0	34.0
Recognition	86.6	27.2
Preparedness	79.6	20.4

Recognition dimension of cultural competence

Table 3 examines the relationship between the recognition dimension of cultural competence and various demographic characteristics. A statistically significant association was found only with specialty (p = 0.02). Family medicine physicians demonstrated the highest recognition scores (mean rank = 47.36), followed by general practitioners (mean rank = 35.10) and physicians from other specialties (mean rank = 19.13). No significant associations were found between recognition scores and other demographic variables, including age, gender, nationality, qualification, or years of experience. While older physicians (46 to 55 years) and those with more experience (20 to 29 years) showed somewhat higher mean ranks for recognition, these differences did not reach statistical significance. It is noteworthy that the physician with 30 or more years of experience had the lowest recognition score (mean rank=1.00); however, this represents only a single physician.

**Table 3:** Association between recognition and demographic characteristics (total n=85) ^a^Kruskal-Wallis H test, ^b^Mann-Whitney U tests, *Significant at p<0.05

Variables		Recognition			
		N	Mean rank	Test statistics	p-value
Age (in years)	25-35	45	44.46	0.99 ^a^	0.61
	36-45	32	39.84		
	46-55	8	47.44		
Gender	Male	30	38.87	701.00 ^b^	0.24
	Female	55	45.25		
Nationality	Saudi	81	43.41	128.50 ^b^	0.48
	Non-Saudi	4	34.63		
Qualification	Bachelors	35	40.44	4.54 ^a^	0.21
	Masters	6	60.50		
	Board certified	39	41.37		
	Subspecialty (fellowship)	5	52.60		
Specialty	General practitioner	21	35.10	8.17 ^a^	0.02*
	Family medicine	60	47.36		
	Other specialty	4	19.13		
Years of experience	1-9	48	42.41	4.56 ^a^	0.21
	10-19	29	42.76		
	20-29	7	54.07		
	≥30	1	1.00		

## Discussion

The findings of the present study revealed that participants generally had a strong recognition of cultural factors and a good understanding of the social and cultural context of patients, but a lower rate of preparedness to address those factors in practice. This means that although physicians appear aware of cultural issues affecting care, they seem to be less equipped to act on that awareness. The only factor that showed a significant association with recognition was specialty, with family medicine physicians scoring highest, followed by general practitioners and then other specialties. No significant differences in recognition were found regarding the participants’ age, gender, nationality, qualification, or years of experience.

The findings of this research align with previous studies. For example, Almuqbil et al. conducted a study on nurses in a tertiary hospital in Saudi Arabia, and they reported a high level of awareness and sensitivity among their participants [16]. Similarly, Alharbi et al. [17] showed that nurses had a high level of cultural competence, with participants having high scores in cultural awareness and performance domains. Thus, our findings can be placed in context with the limited literature on cultural competence in Saudi Arabian healthcare. However, in Saudi Arabia, cultural competence is very relevant. For example, a recent review by Nasr [18] on cultural competence among family medicine practitioners in Saudi Arabia reported that healthcare in Saudi Arabia is increasingly multicultural, and this poses a major challenge in delivering personalized and comprehensive care. Internationally, earlier research has reported lower scores for cultural competence compared to our findings. For example, a cross-sectional study from Turkey [19] reported that the cultural competence score for primary healthcare professionals was 66.58±13.47.

The results of Lin et al.'s [20] study involving Taiwanese nurses support our findings, as they too identified that participants had higher scores for cultural awareness and sensitivity but had low scores regarding cultural skills. Similarly, a study from Finland [21] reported that cross-cultural empathy may protect healthcare professionals from distress and sleep disturbances. The Saudi healthcare environment is characterized by linguistic and cultural diversity, with significant expatriate populations. Cultural competence in healthcare is very important; Kaihlanen et al. [22] pointed out that a lack of cultural understanding increases negative attitudes towards cross-cultural care and affects healthcare professionals’ perceived preparedness to take care of culturally diverse patients. In the present study, age and experience were not significantly associated with recognition. This contrasts with the findings of Almutairi et al. [23], who reported that age was associated with higher levels of cultural competence, as cultural competence requires exposure to caring for people.

Previously, studies have reported that cultural education is crucial in both undergraduate and in-service training to raise competence. However, physician training in cultural competence is far from universal. For example, Mainous et al. [24] analyzed the 2016 United States National Ambulatory Medical Care Survey and found that only 66.3% of physicians reported having any formal cultural competence training. Other studies have also reported these themes. A recent Dutch survey [25] of general practice trainees and trainers found that overall cultural competence was low, especially regarding factual knowledge. About 94% of general practitioner trainees and 81% of general practitioner trainers scored low on cultural knowledge, with nearly half scoring low on attitudes and skills. The researchers noted that cultural competence improved with experience (having >10% migrant patients) and emphasized the need to include cultural competence in training curricula. Similarly, Cruz et al. [26] from Saudi Arabia reported that gender, clinical exposure, and experience with culturally diverse patients were associated with high levels of cultural competence. The evidence shows that cultural competence is a crucial aspect of a healthcare professional, and being aware of one’s own cultural features facilitates cross-cultural encounters [22].

Although the calculated sample size was larger than the available population, we included nearly all eligible physicians, which reduced the risk of sampling bias. Nevertheless, the limited sample size and the focus on a single hospital in Jeddah may restrict the generalizability of findings to other healthcare settings in Saudi Arabia. In addition, self-reporting of the data on cultural competence may be subject to social desirability bias, as participants might overestimate their cultural competence. This study assessed self-reported knowledge, awareness, and preparedness but did not directly measure cultural skills in practice. Future studies should incorporate objective assessments of cultural skills through observed interactions or standardized patient encounters. Furthermore, the study's cross-sectional design restricts the establishment of causal relationships between variables.

Based on our findings, we recommend implementing structured cultural competence training programs that specifically target the preparedness domain, where physicians scored lowest. Healthcare institutions should develop continuing education that focuses on practical skills for culturally appropriate care. Medical education curricula should integrate cultural competence as a core component, emphasizing practical skills alongside knowledge and awareness. Future research should include larger, more diverse samples across multiple Saudi Arabian healthcare institutions to validate these findings. Additionally, we recommend that future studies evaluate physician-patient interactions to provide further insight into cultural competence in practice in other regions of Saudi Arabia. Moreover, randomized clinical trials that assess the impact of cultural competence training programs can guide the health authorities to identify and adopt the most effective training methods.

## Conclusions

Primary healthcare physicians at the KGH demonstrate strong cultural awareness but lack practical implementation skills. Family medicine specialists exhibited higher cultural recognition than other specialties. These findings underscore the importance of targeted cultural competence training, especially in practical communication skills, among primary healthcare physicians. Enhancing cultural preparedness may bridge gaps in patient understanding, improve healthcare outcomes, and support more equitable care delivery in Saudi Arabia’s multicultural society. Policymakers and medical educators should integrate cultural competence into continuing professional development frameworks.

## Figures and Tables

**Figure 1:**
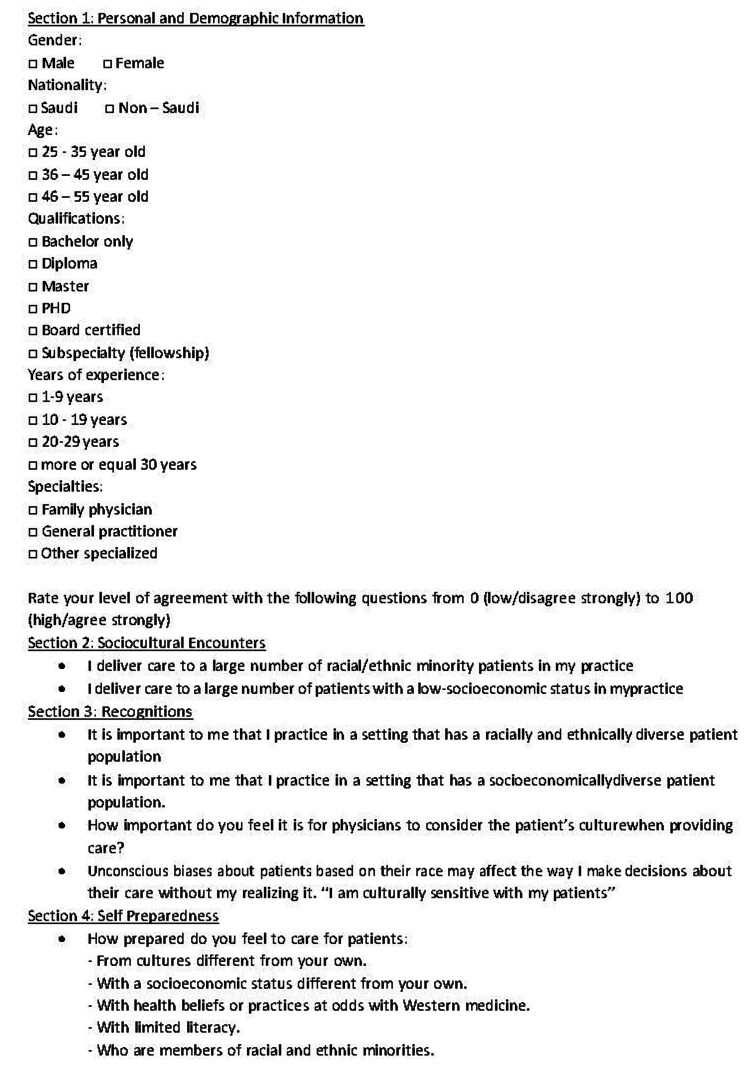
The pre-validated questionnaire used in the current study This questionnaire was originally validated in English by Marshall et al. [5].

## Appendices

Appendix A

Figure 1 features the questionnaire issued to participants of the current study. 
